# The Use of Instagram in the Sports Biomechanics Classroom

**DOI:** 10.3389/fpsyg.2021.711779

**Published:** 2021-08-12

**Authors:** Archit Navandar, Daniel Frías López, Lidia B. Alejo

**Affiliations:** Faculty of Sport Sciences, Universidad Europea de Madrid, Madrid, Spain

**Keywords:** social networks, social media, qualitative analysis, student learning, mobile learning, higher education, teaching, sports science classroom

## Abstract

Students in higher education habitually use mobile devices such as smartphones and tablets, specifically using social networks on them for staying updated on current affairs, communication between one another, and entertainment. However, its use as a potential medium for learning especially in times of distance learning has not been studied in depth. Instagram is the fastest growing social network in the world and its graphical interface makes it a useful learning tool for a theoretical-practical subject such as biomechanics, where movement is analyzed. The main objective was to evaluate the change in perception of students in the use of Instagram as a learning medium for qualitative biomechanical analysis as a part of their undergraduate sports science program. 171 students filled out a questionnaire on the use of smartphones, social networks, and specifically Instagram, before and after doing an assignment of qualitative analysis that was carried. The results indicated a positive change in the perspective of the students when asked if class assignments can be done on mobile devices (*p* = 0.002) and social media (*p* < 0.001). The students also indicated a greater interest in doing assignments via social networks (*p* < 0.001), especially in the subject of biomechanics (*p* < 0.001). They also cited that Instagram is a useful source for information on sports biomechanics (*p* = 0.015) and could be used to make observations of sporting movements (*p* = 0.043). The results indicated that an introduction of familiar devices in undergraduate teaching could produce a positive change in perception of using such methodologies and facilitate learning. The students, who use smartphones very frequently in their daily lives, and specifically, many use them for browsing social networks, find the platform to be very useful in finding and sharing information related to sports and specifically in sports biomechanics. The visual aspect of social networks like Instagram can help engage them with learning strategies in a subject like biomechanics.

## Introduction

Information and Communication Technologies (ICTs) are increasing their influence in the world and have been responsible for bringing about social changes in the last decade (Vilhelmson et al., 2017). Similarly, they are making inroads into the educational field as well, providing an effective environment to facilitate student learning and improving teaching efficiency (Fu, 2013). One of the ubiquitous uses of ICTs has been the use of mobile devices such as smartphones and tablets, and the growing presence of mobile devices in the education environment has greatly shown its potential in promoting its use as an effective learning tool (Khalitova and Gimaletdinova, 2016).

Over the last few years, one of the most important trends in education has been learning through mobile devices (Luguetti et al., 2017) and has been used to perform academic activities ranging from simple activities, such as readings, to more complex activities as oral exhibitions or problem-based learning (Tang and Hew, 2017). This use has been further enhanced presently with the lockdown caused by the COVID-19 pandemic causing face-to-face teaching to move online overnight (Knudson, 2020). All around the world, owing to economic and social constraints, students have not had access to traditional PCs and have had to follow lessons on mobile devices (Biswas et al., 2020; Rodriguez Martinez, 2020; The Hindu, 2020), resulting in the increase in the downloads of education-centric apps and greater time spent on these devices (Ohme et al., 2020).

Students in higher education habitually use mobile devices such as smartphones and tablets (Gikas and Grant, 2013), specifically using social networks on them for staying updated on current affairs, communication between one another, and entertainment (Chaffey, 2018). Mobile devices, through social networks, provide immediacy and openness, enabling them to be used both as an information source and as a collaborative tool (Junco et al., 2013), and hence its use in the educational environment must be considered (Piñón et al., 2016) as their use is also on the rise (Wiley, 2019).

A survey conducted just before the COVID-19 pandemic showed that 49% of the global population uses social networks (Kemp, 2020), with this percentage being higher in developed countries where there was greater access to mobile devices (The Social Media Family, 2018; Kemp, 2020). Specifically, social networks were popular in people aged between 18 and 39 years (The Social Media Family, 2018) with those belonging to this age group being known as “digital natives” (Boyd, 2007).

Social networks are gradually being used as a novel tool for teaching and learning (Lescano and Quiroga, 2017), facilitating the development of student-professor and student-student interactions (Evans, 2014), and increasing the scope of the learning environment by permitting easy interaction between people from all over the world in different and innovative ways (Bista, 2015). Studies using social networks as an educational tool have shown a positive impact on learning research, demonstrating that social networks could improve student learning, affective learning, creativity (Dawson et al., 2011), and classroom climate, both inside and outside the class (Brady et al., 2010). In the context of higher education, many studies have analyzed the relationship between the use of social networks, such as Twitter (Tang and Hew, 2017), Facebook (Hew, 2011; Cuesta et al., 2015), and YouTube (Roodt and Peier, 2013) as a didactic tool and its possible benefits in learning, however, there are only a few studies which have used Instagram (Manca, 2020).

Instagram is the fastest growing social network in the world (The Social Media Family, 2018) with over one billion active users (Kemp, 2020) and is the most popular social network amongst millennials (Kemp, 2020). Instagram is a mobile-centric photo and video capturing and sharing platform that allows one to tag, and search for images and videos using hashtags (Sheldon and Bryant, 2016). Given that it is a more visual platform, it has the potential to be a great learning medium considering that there is previous research which states that students retain information better when it is presented visually (Levie and Lentz, 1982; Fisk et al., 1994; Kizilcec et al., 2014). Moreover, this graphical interface of Instagram could be useful in facilitating learning in biomechanics, a subject that is essentially based on the analysis of movement.

Undergraduate biomechanics courses teach human movement through principles of classical mechanics, motor control, neural control, and anatomy (Knudson et al., 2009) and their relation to physical function and mobility in activities of daily living, sports, and pathological conditions (Riskowski, 2015). Biomechanics instructors surveyed in North America highlighted the importance of kinematic analysis as a fundamental part of the curriculum; but also showed that the biggest challenges they face in classes of introductory biomechanics are the difficulties associated with learning math (60% of the respondents) and physics (27% of the respondents) (Garceau et al., 2012), both of which form the crux of kinematic analysis. Although they suggest a preparatory course or easier access to biomechanical equipment, the limited economic and time resources for the faculty and the heterogeneity of students’ backgrounds make implementing such strategies difficult (Garceau et al., 2012).

Different approaches using blended (Riskowski, 2015) and active learning (Knudson, 2020) have been suggested to engage undergraduate biomechanics students, but a study has shown that these approaches might face difficulty when students are not sufficiently autonomous to undertake independent study (Keogh et al., 2017). With remote learning enforced by the COVID-19 pandemic, approaches such as blended and active learning might be difficult to implement remotely, but an audio-visual platform such as Instagram has the potential to overcome such hurdles. Since qualitative analysis forms, the basis of any biomechanical analysis in sport (Knudson, 2007a), the use of technology (that they are familiar with) could be a possible approach to help students into learning biomechanical concepts and alleviating the fears of math and physics temporarily. Therefore, the present study has the main objective of evaluating the change in perception of students in the use of Instagram as a learning medium for qualitative biomechanical analysis as a part of their undergraduate sports science program. The hypothesis was that the use of Instagram would change their perceptions positively after performing the designated assignment.

## Materials and Methods

### Study Design

An experimental study design was applied where students filled out questionnaires before and after undertaking an assignment on sports biomechanics. Approval for the research design was obtained from the ethics committee of the Universidad Europea de Madrid.

### Participants

An *a priori* power analysis was performed with a 95% confidence interval and Cohen’s *d* = 0.3, power of 0.95, giving the required sample to be composed of at least 154 participants (Faul et al., 2009). 243 students enrolled in the sports biomechanics course (2nd year of the undergraduate sports science program) were invited to participate in this study, of which 171 undergraduate students in sport sciences (males: *n* = 141; females: *n* = 30) aged 21.98 ± 3.39 years (range: 19–48 years) finally volunteered to participate in this study. This was the only biomechanics course they had over the four-year study program, and the course consisted of 72 h of face-to-face learning, with each session lasting no more than 100 min. The students that participated gave their consent to their participation in the research study.

### Assignment

As a part of the assignment, the students had to carry out a qualitative biomechanical analysis of a sports movement of their choice, basing their analysis on the guidelines indicated by Knudson (2007a) and previously taught in class. The explanation of qualitative analysis was done over two theoretical-practical classes, where they were taught about how to develop a structured framework of analysis (Bartlett, 1997). This required them to follow the four stages of biomechanical analysis (Knudson and Morrison, 2002; Knudson, 2007b): preparation, observation, evaluation and diagnosis, and intervention. Emphasis was given to the phases of preparation and observation, and the students submitted an initial needs analysis, establishing critical features of the movement, through the Blackboard^®^ virtual learning environment (Blackboard Inc., Washington D.C., United States).

The students had three weeks to submit the entire assignment as a single post on Instagram which involved the four stages of qualitative analysis. For this part of the assignment, the students were required to create a private account on Instagram (Kassens, 2014). They were free to use relevant emojis, hashtags, other tools offered by Instagram present at that time (like Super Zoom, Rewind, etc.), and could use other applications and software to prepare their post as long as it did not exceed the restrictions of a single post on Instagram (a maximum of ten photos and/or videos per post, with a video not lasting over 59 s, Instagram TV was not an available feature at the time of implementing the research). During these three weeks, the students could clarify their doubts about qualitative biomechanical analysis or the use of Instagram with the professor.

### Questionnaire

First, an initial questionnaire was designed with forty questions and sent to fifteen experts for their validation. The experts were professionals in the educational sector and taught sports biomechanics, research methods, and pedagogy-related subjects. The initial questionnaire was sent to them via Google Forms and their responses were recorded online anonymously, and the experimental design was explained to them via telephone and/or email. They had to rate the questions on relevance on a five-point Likert scale, with 1 representing “not relevant” and 5 representing “very relevant.” The experts also had an option of open-ended comments that they could leave to give their overall feedback on the questionnaire. Finally, twelve experts (females = 6, males = 6; age: 39.11 ± 8.14 years; 9 with PhDs and 3 with Master’s degrees; Teaching experience = 12.33 ± 8.01 years; all used some social networks and ten had Instagram accounts). Once the results were obtained from the experts, the coefficient of content validation validity of the questionnaire was calculated using Aiken’s V, and its 95% confidence intervals were also determined (Penfield and Giacobbi, 2004). Only those questions were selected that had a content validity score of 0.75 or more (Penfield and Giacobbi, 2004). The calculations were carried out using Microsoft Excel 2016 software (Microsoft^®^, Redmond, United States). Finally, a questionnaire of the twenty-seven validated questions (reliability of 93.3%, Table 1) was used for the study (Table 1).

**TABLE 1:** The questionnaire passed to the students representing the type of question and the section to which it belonged. For each question, the Aiken’s V validity score (along with its 95% confidence interval (CI)) and its reliability are shown.

N°.	Question	Type of response	Section	V-Aiken	Lower CI (95%)	Upper CI (95%)	Reliability (%)
1	How long do you use your smartphone in a day?	Multiple-Responses	Smartphones	0.75	0.6	0.86	86.4%
2	Which is the most used app on your smartphone?	Open		1	0.91	1	100%
3	What are the three main reasons for using mobile phones (in order)?	Open		0.75	0.6	0.86	90.9%
4	Can mobiles and tablets replace traditional books?	Likert		0.9	0.76	0.96	92.7%
5	Can mobiles replace PCs?	Likert		0.9	0.76	0.96	87.3%
6	Can you study through your mobile device?	Likert		0.85	0.71	0.93	92.7%
7	Is the mobile/smartphone a good tool to search for information?	Likert		0.83	0.69	0.92	94.5%
8	Can classroom assignments be done exclusively on the mobile/smartphone?	Likert		0.83	0.69	0.92	94.5%
9	How long do you use you spend on social networks?	Multiple-Responses	Social Networks	0.88	0.74	0.95	90.9%
10	Indicate the social networks that you use	Open		0.96	0.85	0.99	90.9%
11	What are the main reasons for using social networks?	Open		0.79	0.64	0.89	100%
12	Do you think that social networks are a good source of sporting information?	Likert		0.96	0.85	0.99	92.7%
13	Can you do classroom assignments through social networks?	Likert		0.92	0.79	0.97	96.4%
14	Do you think that the use of social networks can generate too much information?	Likert		0.75	0.6	0.86	94.5%
15	Do you think that social networks distract students instead of aiding them?	Likert		0.9	0.76	0.96	94.5%
16	Do you think that social networks play an important role in the sports field today?	Likert		0.79	0.64	0.89	92.7%
17	Would you like to do class assignments on social networks?	Likert		0.92	0.79	0.97	94.5%
18	Are you worried about privacy on social networks?	Likert		1	0.91	1	96.4%
19	Do you have an Instagram account?	Yes/No	Instagram	0.92	0.79	0.97	100%
20	How often do you check Instagram?	Multiple-Responses		1	0.91	1	86.4%
21	Can the use of social networks be prejudiced against introverted students?	Likert		0.75	0.6	0.86	87.3%
22	Can Instagram be used as a source of sporting information?	Likert		0.88	0.74	0.95	96.4%
23	How important is Instagram in the sports field today?	Likert		0.85	0.71	0.93	92.7%
24	Can Instagram be a useful source of information on Sports Biomechanics?	Likert		0.9	0.76	0.96	96.4%
25	Can Instagram tools be useful to make observations of sporting movements?	Likert		1	0.91	1	98.2%
26	How important is Instagram in reaching clients in the sports field?	Likert		0.88	0.74	0.95	94.5%
27	Can biomechanics assignments be done on Instagram instead of the Virtual Environment (Blackboard)?	Likert		0.94	0.82	0.98	89.1%

Students filled out online questionnaires (Table 1) before and after the assignment through a link sent to them by the professor. The pre-assignment questionnaire included questions divided into four different categories: (1) the use of smartphones and using a smartphone as a learning medium; (2) the use of social networks and using social networks as learning mediums; and (3) the use of Instagram and using it as a learning platform (only the students who had Instagram accounts answered these questions). The questions consisted of open questions, closed multiple responses, and a five-point Likert-type scale, where 1 represented “complete disagreement with the statement,” and 5 represented “complete agreement with the statement.”

### Statistical Analysis

The responses from all participants were downloaded as a spreadsheet and responses from the open-ended questions were grouped. Since the responses were anonymous, they were grouped as pre- and post-assignment responses and input into Tableau (version 2020.3, Salesforce, Washington, Seattle) for analysis and visualization. The data from the first section of the questionnaire (the use of smartphones and social networks) with open responses were represented as packed bubble charts, and pie charts were used to represent yes/no questions. The responses from sections two, three, and four (using smartphones/social networks/Instagram as learning mediums) were represented in a Gantt chart, with each response being given a specific color, and the mean was represented as a black dot. The size of the bubble/Gantt bar represented the number of people that responded with the corresponding answer.

The responses to the nineteen Likert-scale type questions were compared pre- and post-assignment using non-parametric tests. Significance set at α = 0.05, and effect sizes of the differences were determined using Cohen’s *d*, with the following thresholds being used to interpret the findings: 0.2 ≤ *d* < 0.5 small differences; 0.5 ≤ *d* < 0.8 medium differences; and *d* ≥ 0.8 large differences. All statistical analysis was carried out on Jamovi (version 1.8.1^1^).

## Results

**FIGURE 1:**
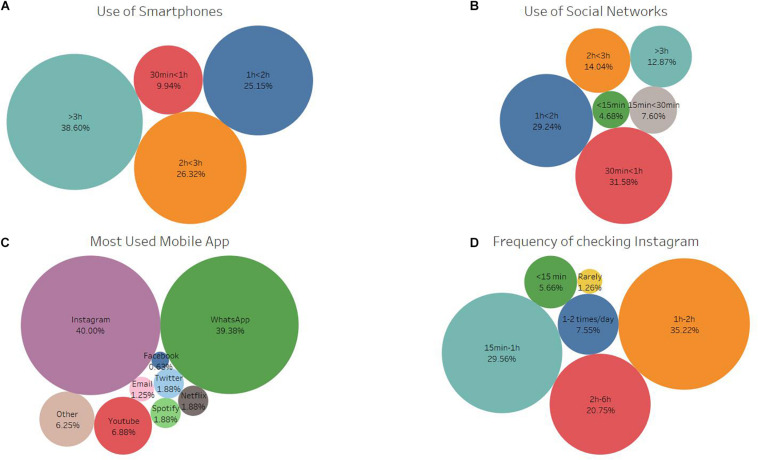
The participants’ self-reported use of smartphones and social networks. **(A,B)** The daily time spent on smartphones and social networks. **(C)** The most used app on their smartphone. **(D)** The frequency of checking Instagram on a daily basis.

**FIGURE 2:**
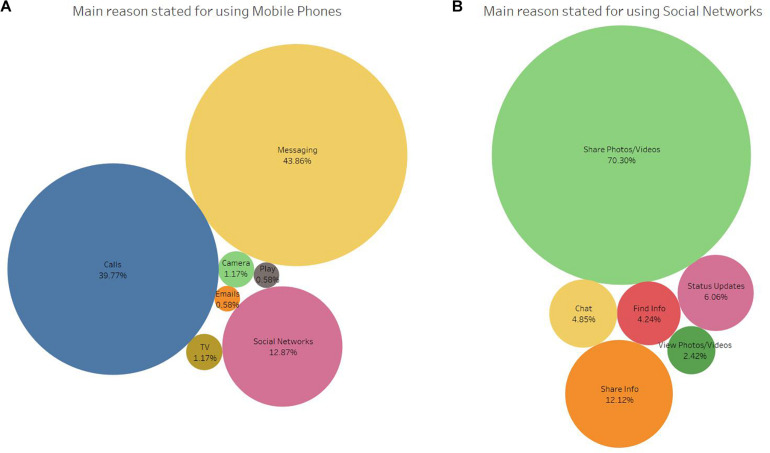
The participants’ self-reported, main reason for using mobile phones **(A)** and social networks **(B)**.

**FIGURE 3:**
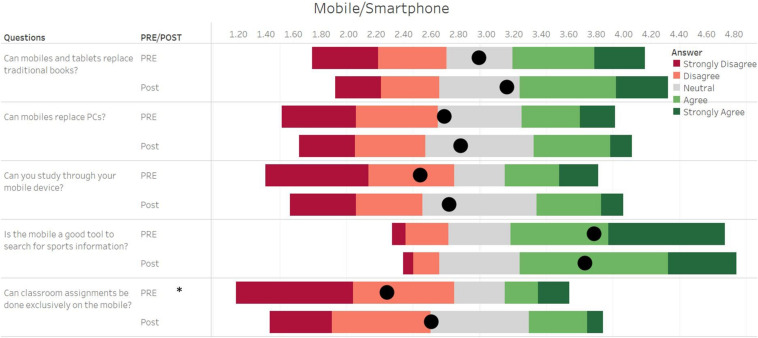
Student responses for questions in the survey related to smartphone usage. The length of each bar of the Gantt chart represents the number of corresponding responses before (PRE) and after (Post) activity, with the black dot representing the average of all values. The responses were recorded on a five-point Likert-type scale, where 1 represented a strong disagreement with the question and 5 represented a complete agreement to the question. * Represents a significant difference between pre- and post-values at *p* < 0.05.

**FIGURE 4:**
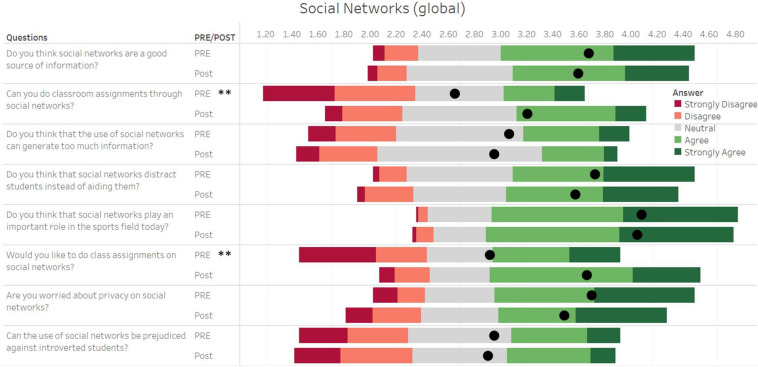
Student responses for questions in the survey related to social networks. The length of each bar of the Gantt chart represents the number of corresponding responses before (PRE) and after (Post) activity, with the black dot representing the average of all values. The responses were recorded on a five-point Likert-type scale, where 1 represented a strong disagreement with the question and 5 represented a complete agreement to the question. ** Represents a significant difference between pre- and post-values at *p* < 0.001.

**FIGURE 5:**
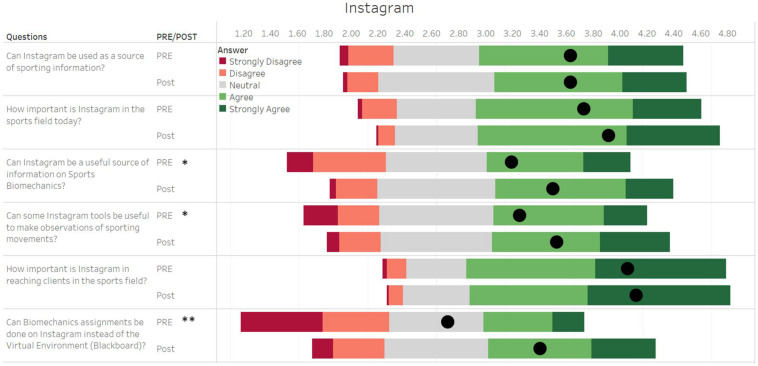
Student responses for questions in the survey related to Instagram. The length of each bar of the Gantt chart represents the number of corresponding responses before (PRE) and after (Post) activity, with the black dot representing the average of all values. The responses were recorded on a five-point Likert-type scale, where 1 represented a strong disagreement with the question and 5 represented a complete agreement to the question. * Represents a significant difference between pre- and post-values at *p* < 0.05. ** Represents a significant difference between pre- and post-values at *p* < 0.001.

The main results from the pre- and post-assignment questionnaires are shown in Figures 1–5, and the number of respondents per answer is indicated by the size of the bars.

### Usage of Smartphones, Social Networks, and Instagram

Instagram was the most popular social network with 93.57% of the participants using it, compared to 41.52% for Facebook, 35.67% for Twitter, and 5.85% for Snapchat.

The daily usage of smartphones (Figure 1A) showed that 38.60% used their phones for over 3 h, 26.32% between 2 and 3 h, 25.15% between 1 and 2 h, and 9.94% under 1 h. When comparing this to the use of social networks (Figure 1B), 12.87% of the participants admitted to spending over 3 h on them, 14.04% between 2 and 3 h, 29.24% between 1 and 2 h, while 43.86% used them for under an hour. Instagram (37.43%) and WhatsApp (36.84%) were the most used applications (Figure 1C). Specifically, in the case of Instagram, 32.75% checked the application every 1–2 h, 27.49% every 15 min–1 h, and 19.30% between 2 and 6 h (Figure 1D).

Instant messaging (43.86%) and “making/receiving calls” (39.77%) were cited by the participants as the most important reason for using their smartphones (Figure 2A). Sharing photos/videos were cited by most of the participants (67.84%) as the reason for using social networks (Figure 2B), followed by sharing information (11.70%) and posting status updates (5.85%).

### Comparing Perceptions Before and After the Assignment

#### Smartphones

Comparing pre- and post-data on the questionnaire (Figure 3), significant differences were obtained for the question “*Can classroom assignments be done exclusively on mobile devices?*” (PRE: 2.28 ± 1.29 vs POST: 2.60 ± 1.12; *p* = 0.002, *d* = 0.33). Small differences were observed for the question “*Can you study through your mobile device?*,” but this result was statistically non-significant (PRE: 2.52 ± 1.38 vs POST: 2.73 ± 1.18; *p* = 0.070, *d* = 0.20). No other significant differences were observed for the questions asked (Figure 3).

#### Social Networks

Significant differences were observed for the questions “*Can you do classroom assignments through social networks?*” (PRE: 2.65 ± 1.25 vs POST: 3.20 ± 1.03; *p* < 0.001, *d* = 0.49) and “*Would you like to do classroom assignments on social networks?*” (PRE: 2.92 ± 1.41 vs POST: 3.66 ± 1.08; *p* < 0.001, *d* = 0.58). The other questions related to the use of social networks showed no significant differences when comparing data pre- to post-assignment (Figure 4).

#### Instagram

Three questions asked on the Instagram section of the questionnaire showed small to medium significant differences when comparing pre- and post-assignment data (Figure 5): “*Can Instagram be a useful source of information on sports biomechanics?*” (PRE: 3.19 ± 1.15 vs POST: 3.63 ± 0.93; *p* = 0.015, *d* = 0.27); “*Can some Instagram tools be useful to make observations of sporting movements?*” (PRE: 3.25 ± 1.14 vs POST: 3.53 ± 1.06; *p* = 0.043, *d* = 0.23); and “*Can biomechanics assignments be done on Instagram instead of the Blackboard virtual environment?*” (PRE: 2.72 ± 1.29 vs POST: 3.40 ± 1.13; *p* < 0.001, *d* = 0.55). The other questions showed no significant differences.

## Discussion

This study aimed to look at the changes in perception of using Instagram in the teaching of the undergraduate introductory course of biomechanics. 171 students filled out a questionnaire on the use of smartphones, for social networks and specifically on Instagram before and after doing an assignment of qualitative analysis that they presented via Instagram. The results confirmed the hypothesis and indicated a positive change in perception in using Instagram to submit the qualitative analysis assignment, and that an introduction of familiar devices and tools from day-to-day life in teaching could facilitate learning in conjunction with traditional teaching methods.

There was a large change in perspective in the participants about the use of social networks to do assignments both in general and specifically in the subject of biomechanics (Figures 3–5). Instagram helped them carry out the task easily as indicated by the results in which they preferred using Instagram to a traditional virtual learning environment (Figure 5). These results were similar to those seen in students of radiology (Shafer et al., 2018), where the students learned more by tasks on Instagram as compared to traditional learning environments. The study of Sugimoto et al. (2015) further corroborates the finding that the use of social networks in the classroom could enhance learning experiences in undergraduate students. Given the limited time on hand per subject in the undergraduate degrees, biomechanics teachers tend to lay lesser importance on the qualitative analysis of movement (Garceau et al., 2012). Moreover, they often cite the fear of math and physics as the biggest hurdles in teaching (Garceau et al., 2012). The introduction of qualitative movement analysis in a familiar environment for the students such as Instagram could address this issue and further helped students before delving into math and physics-centric kinematic and kinetic analysis.

As of today, Instagram is by far the most popular social network in the age group of students, the results of this study reconfirm the statistics seen worldwide (Kemp, 2020). Instagram, a social network that uses audio-visual information, can serve as an excellent support learning tool, without completely replacing traditional teaching methods. Learning through Instagram moves away from compiling information and contents of the subjects in written form by setting up communication channels, which often occur in a non-hierarchical and informal context, and encourage bidirectional communication (Burns et al., 2016). This is especially crucial when access to laboratories and/or equipment may be restricted, such as in the case of distance learning. Instagram can bridge the gap by not only helping to make useful observations of sporting movements as indicated by the results of the study (Figure 5), but also open communication channels between students and teachers, and with other practitioners (biomechanists, researchers, sports scientists, coaches, athletes, etc.) beyond the walls of the traditional classroom.

The results of the study reinforce this point as after doing the assignment, the students found Instagram to be a useful source of information for sports biomechanics (Figure 5). In the long term, such sources can help students contribute more to online communities of their interest when they feel comfortable with the environment and learn from the contribution of others (Akhiar et al., 2017). Given that the students use the application very frequently (Figure 1), Instagram can facilitate the active participation of students and can mediate the process of self-learning (Khalitova and Gimaletdinova, 2016). This can potentially address the situation of students from heterogeneous backgrounds, with each student being able to find information that is specific to the student’s field of interest. Moreover, this can also benefit the students who are shy to participate in face-to-face classes by allowing them to participate in communication without relying on social trust (Burns et al., 2016), as they can “like,” “comment,” and “share” content that is not their own (Mikum et al., 2018).

The overall results of this study indicated that the students participated more using Instagram for their assignments, a finding similar to that previously reported in the literature (Hortigüela-Alcalá et al., 2019). The use of Instagram could increase student participation (Hortigüela-Alcalá et al., 2019). Both these stem from the familiarity and simplicity of the tools used, along with Instagram’s quick access through mobile devices and the application’s attractive interface (Khalitova and Gimaletdinova, 2016). Social networks increase the ease of transmitting information through very small devices, and the simpler the application, the more profitable its use will be and probably more students will use it to participate (Mikum et al., 2018), making this aspect tangible in the case of Instagram.

It is important to remark that the use of Instagram as a learning tool has its inconveniences as well. Students seem to be averse to learning through mobile devices and are not convinced that a smartphone, as of today, can completely replace a PC or the knowledge of traditional books (Figure 3). However, this study was carried out before the onset of the COVID-19 pandemic, and it would certainly be interesting to replicate this study now in order to gage the impact of the pandemic in changing these perceptions. Although the students might be well-versed with the use of digital tools, they are still not used to using social networks educationally (Akhiar et al., 2017). The use of social networks in teaching is still in its nascent stage and as a learning tool, it must not detract students from conventional teaching (Burns et al., 2016), rather complement it.

The primary purpose of teaching within the field of biomechanics is to develop the cognitive and practical knowledge required to function as competent practitioners within the chosen field (Riskowski, 2015), and Instagram could perhaps provide that platform. This platform can be used as a steppingstone to introduce basic concepts of movement analysis, involve students based on their varied interests, before delving into the introduction of more complex kinematic and kinetic analysis concepts. Thus, students are exposed to real-world applications of biomechanics while simultaneously addressing the issues of limited space and/or equipment (Garceau et al., 2012). Nevertheless, it is important to highlight the lessons taught with them must be well-planned (Grosseck, 2009) using the right methodologies (Hamid et al., 2009) keeping in mind the diversity of the students. Considering that the task required a simple video analysis, the fact that the theoretical base was previously taught over the course of two lessons (200 min), making the use of Instagram easier for the task assigned.

Given the situation that the world is passing through, as professors, we have had to move to remote learning overnight. In this regard, social networks can be very useful for engaging the biomechanics community, as evidenced by the popularity of the *Sports Biomechanics Lecture Series* (McErlain-Naylor, 2020), but to encourage future students into biomechanics, one must speak their language. Although the results of the study are promising, one must consider the limitations of this study. This study focused solely on the qualitative aspect of sports biomechanics lectures. Future studies can look at incorporating quantitative analysis, which also forms an important part of biomechanics studies. Secondly, this study looked at only the sports biomechanics subject, and professors can look at using social networks in other subjects of sports science to gage its feasibility in the overall sports science course. Thirdly, the study only analyzed the Likert scale scores of qualitative responses and incorporating studies that contextualize actual student feedback will help improve the application of social networks in the higher education setting. Finally, based on the recommendations of the ethics committee, the students created private accounts for this study – the results of using public accounts, where social networks provide analytics describing the reach of the account and each publication can help the students understand user engagement in a particular social network and benefit from it when they work in a professional environment.

More studies are needed which use on the use of social networks in teaching, and it is important to get both qualitative feedback from the students in terms of their experiences and simultaneously gage the learning that has occurred. These studies ought to be robust statistically analyzed using big samples, but practically this might be difficult to carry out in a single university. Although there is resistance to the use of social networks in education, they have the potential of being used to facilitate learning with an excellent effect. In the next few years, Instagram will likely be replaced by another social network, or maybe Instagram is not the most popular social network in the country of the reader, nevertheless, it is expected that the aspects extracted from this study can be considered for future applications.

## Conclusion

The results show that the perception of undergraduate students in using Instagram to learn qualitative biomechanical analysis is quite positive. The students, who use smartphones very frequently in their daily lives, and specifically, many use them for browsing social networks, find the platform to be very useful in finding and sharing information related to sports and specifically in sports biomechanics. The students, after performing the assignment, did not only believe that they could use smartphones, and social networks in particular, to perform classroom assignments, but also were more willing to use it in their studies. They also believed that Instagram in particular could be a useful source of information on sports biomechanics, and could also be a platform to present their work. This could be attributed to the visual aspect of social networks like Instagram which can help engage them with learning strategies in a subject like biomechanics. Given the large sample of students that participated in the study, the results show a possible positive application of social networks in education and specifically in sports education.

## Data Availability Statement

The original contributions presented in the study are included in the article/supplementary material, further inquiries can be directed to the corresponding author.

## Ethics Statement

The studies involving human participants were reviewed and approved by Comité de Investigación, Universidad Europea de Madrid. The participants provided their written informed consent to participate in this study.

## Author Contributions

AN organized the database and performed the statistical analysis. All authors wrote the first draft of the manuscript and contributed to the conception, design of the study, manuscript revision, read, and approved the submitted version.

## Conflict of Interest

The authors declare that the research was conducted in the absence of any commercial or financial relationships that could be construed as a potential conflict of interest.

## Publisher’s Note

All claims expressed in this article are solely those of the authors and do not necessarily represent those of their affiliated organizations, or those of the publisher, the editors and the reviewers. Any product that may be evaluated in this article, or claim that may be made by its manufacturer, is not guaranteed or endorsed by the publisher.

## Footnotes

1
www.jamovi.org

## References

[B1] Akhiar A. Mydin A.-A. Kasuma S. A. A. (2017). Students’ perceptions and attitudes towards the use of Instagram in English language writing. *Education* 47–72.

[B2] Bartlett R. (1997). *Introduction to sports biomechanics.* Oxfordshire: Routledge.

[B3] Bista K. (2015). Is Twitter a pedagogical tool in higher education? Perspectives of education graduate students. *J Scholarsh. Teach. Learn.* 15 83–102. 10.14434/josotl.v15i2.12825

[B4] Biswas B. Roy S. K. Roy F. (2020). Students perception of Mobile Learning during COVID-19 in Bangladesh: university student perspective. *Aquademia* 4:ep20023. 10.29333/aquademia/8443

[B5] Boyd D. (2007). Social network sites: public, private, or what. *Knowledge tree* 13 1–7. 10.1016/b978-075068054-7.50025-3

[B6] Brady K. P. Holcomb L. B. Smith B. V. (2010). The use of alternative social networking sites in higher educational settings: a case study of the e-learning benefits of Ning in education. *J. interact. Online Learn.* 9 1541–4914.

[B7] Burns E. Rees J. C. Maclachlan J. (2016). Everybody phones out: teaching experiments with Instagram. *Spark: UAL Creative Teaching and Learning Journal* 1 79–94.

[B8] Chaffey D. (2018). *Global social media research summary 2018 [Online]. SmartInsights.* Available online at: https://www.smartinsights.com/social-media-marketing/social-media-strategy/new-global-social-media-research/ (accessed May 15, 2021).

[B9] Cuesta M. Eklund M. Rydin I. Witt A.-K. (2015). Using Facebook as a co-learning community in higher education. *Learn. Media Technol.* 41 55–72. 10.1080/17439884.2015.1064952

[B10] Dawson S. Tan J. P. L. Mcwilliam E. (2011). Measuring creative potential: using social network analysis to monitor a learners’ creative capacity. *Australas. J. Educ. Technol.* 27 924–942.

[B11] Evans C. (2014). Twitter for teaching: can social media be used to enhance the process of learning. *Br. J. Educ. Technol.* 45 902–915. 10.1111/bjet.12099

[B12] Faul F. Erdfelder E. Buchner A. Lang A.-G. (2009). Statistical power analyses using G^∗^ Power 3.1: tests for correlation and regression analyses. *Behav. Res. Methods* 41 1149–1160. 10.3758/brm.41.4.1149 19897823

[B13] Fisk A. D. Hertzog C. Lee M. D. Rogers W. A. Anderson-Garlach M. (1994). Long-term retention of skilled visual search: do young adults retain more than old adults? *Psychol. Aging* 9 206–215. 10.1037/0882-7974.9.2.206 8054168

[B14] Fu J. (2013). Complexity of ICT in education: a critical literature review and its implications. *Int. J. Educ. Dev. Using ICT* 9 112–125.

[B15] Garceau L. R. Ebben W. P. Knudson D. V. (2012). Teaching practices of the undergraduate introductory biomechanics faculty: a North American survey. *Sports Biomech.* 11 542–558. 10.1080/14763141.2012.725764 23259243

[B16] Gikas J. Grant M. M. (2013). Mobile computing devices in higher education: student perspectives on learning with cellphones, smartphones & social media. *Internet High. Educ.* 19 18–26. 10.1016/j.iheduc.2013.06.002

[B17] Grosseck G. (2009). To use or not to use web 2.0 in higher education? *Procedia Soc. Behav. Sci.* 1 478–482.

[B18] Hamid S. Chang S. Kurnia S. (2009). Identifying the use of online social networking in higher education. *Same places, different spaces. Proceedings ascilite Auckland* 419–422.

[B19] Hew K. F. (2011). Students’ and teachers’ use of Facebook. *Comput. Hum. Behav.* 27 662–676. 10.1016/j.chb.2010.11.020

[B20] Hortigüela-Alcalá D. Sánchez-Santamaría J. Pérez-Pueyo Á Abella-García V. (2019). Social networks to promote motivation and learning in higher education from the students’ perspective. *Innov. Educ. Teach. Int.* 56 412–422. 10.1080/14703297.2019.1579665

[B21] Junco R. Elavsky C. M. Heiberger G. (2013). Putting twitter to the test: assessing outcomes for student collaboration, engagement and success: twitter collaboration & engagement. *Br. J. Educ. Technol.* 44 273–287. 10.1111/j.1467-8535.2012.01284.x

[B22] Kassens A. L. (2014). Tweeting your way to improved #writing, #reflection, and #community. *J. Econ. Educ.* 45 101–109.

[B23] Kemp S. (2020). *DIGITAL 2020, GLOBAL DIGITAL OVERVIEW. Essential insights into how people around the world use the internet, mobile devices, social media, and ecommerce.* Available online at: https://wearesocial.com/blog/2020/01/digital-2020-3-8-billion-people-use-social-media (accessed November 13, 2020).

[B24] Keogh J. W. L. Gowthorp L. Mclean M. (2017). Perceptions of sport science students on the potential applications and limitations of blended learning in their education: a qualitative study. *Sports Biomech.* 16 297–312. 10.1080/14763141.2017.1305439 28534436

[B25] Khalitova L. Gimaletdinova G. (2016). Mobile technologies in teaching English as a foreign language in higher education: a case study of using mobile application Instagram. *Proceedings 9th International Conference of Education, Research, and Innovation* 1, 6155–6161.

[B26] Kizilcec R. F. Papadopoulos K. Sritanyaratana L. (2014). Showing face in video instruction: effects on information retention, visual attention, and affect. *Proceedings of the SIGCHI conference on human factors in computing systems* 2095–2102.

[B27] Knudson D. (2007a). *Fundamentals of biomechanics.* New York, NY: Springer Science.

[B28] Knudson D. (2007b). Qualitative biomechanical principles for application in coaching. *Sports Biomech.* 6 109–118. 10.1080/14763140601062567 17542182

[B29] Knudson D. (2020). A tale of two instructional experiences: student engagement in active learning and emergency remote learning of biomechanics. *Sports Biomech.* 14 1–11. 10.1080/14763141.2020.1810306 32924795

[B30] Knudson D. Morrison C. (2002). *Qualitative Diagnosis of Human Movement*, 3rd Edn. Champaign, IL: Human kinetics.

[B31] Knudson D. Bauer J. Bahamonde R. (2009). Correlates of learning in introductory biomechanics. *Percept. Mot. Skills* 108, 499–504. 10.2466/pms.108.2.499-504 19544954

[B32] Lescano M. P. Quiroga D. O. (2017). *#ProblemasParaPensar: inspirar, motivar y sugerir aprendizajes. Proceedings of IV Jornadas de TIC e Innovación en el Aula (La Plata, Argentina).* Available online at: http://sedici.unlp.edu.ar/handle/10915/65766 (accessed July 28, 2021).

[B33] Levie W. H. Lentz R. (1982). Effects of text illustrations: a review of research. *ECTJ* 30 195–232. 10.1007/bf02765184

[B34] Luguetti C. Goodyear V. A. André M. H. (2017). That is like a 24 hours-day tournament!’: using social media to further an authentic sport experience within sport education. *Sport Educ. Soc.* 24, 78–91. 10.1080/13573322.2017.1292235

[B35] Manca S. (2020). Snapping, pinning, liking or texting: investigating social media in higher education beyond Facebook. *Internet High. Educ.* 44:100707. 10.1016/j.iheduc.2019.100707

[B36] McErlain-Naylor (2020). *Sports Biomechanics Lecture Series.* Available online at: https://www.youtube.com/c/StuartMcErlainNaylor (accessed July 28, 2021).

[B37] Mikum S. Suksakulchai S. Chaisanit S. Murphy E. (2018). Students’ participation in peer-to-peer communication supported by social media. *Educ. Inf. Technol.* 23 659–679. 10.1007/s10639-017-9628-8

[B38] Ohme J. Abeele M. M. P. V. Van Gaeveren K. Durnez W. De Marez L. (2020). Staying Informed and Bridging “Social Distance”: Smartphone News Use and Mobile Messaging Behaviors of Flemish Adults during the First Weeks of the COVID-19 Pandemic. *Socius* 6 1–14. 10.1177/2378023120950190 PMC7472194 34192139

[B39] Penfield R. D. Giacobbi J. P. R. (2004). Applying a score confidence interval to Aiken’s item content-relevance index. *Meas. Phys. Educ. Exerc. Sci.* 8 213–225. 10.1207/s15327841mpee0804_3

[B40] Piñón L. Sapién A. Gutierrez M. C. (2016). Social Networks in University Students: Academic Use and Learning Scenarios. *Int. Rev. Manag. Bus. Res.* 5 1039–1047.

[B41] Riskowski J. L. (2015). Teaching Biomechanics with Just-in-Time Teaching (JiTT). *Sports Biomech.* 14 168–179. 10.1080/14763141.2015.1030686 25952410

[B42] Rodriguez Martinez M. (2020). *El móvil salva la brecha escolar en tiempos de cuarentena por el coronavirus [The mobile bridges the school gap in times of quarantine due to the coronavirus].* Lyon: Euronews.

[B43] Roodt S. Peier D. (2013). Using YouTube© in the classroom for the net generation of students. *Proceedings of the Informing Science and Information Technology* 10 473–488. 10.28945/1823

[B44] Shafer S. Johnson M. B. Thomas R. B. Johnson P. T. Fishman E. K. (2018). Instagram as a vehicle for education: what radiology educators need to know. *Acad. Radiol.* 25 819–822. 29751861 10.1016/j.acra.2018.03.017

[B45] Sheldon P. Bryant K. (2016). Instagram: motives for its use and relationship to narcissism and contextual age. *Comput. Hum. Behav.* 58 89–97. 10.1016/j.chb.2015.12.059

[B46] Sugimoto C. Hank C. Bowman T. Pomerantz J. (2015). Friend or faculty: social networking sites, dual relationships, and context collapse in higher education. *First Monday* 20. 10.5210/fm.v20i3.5387

[B47] Tang Y. Hew K. F. (2017). Using Twitter for education: beneficial or simply a waste of time? *Comput Educ.* 106 97–118. 10.1016/j.compedu.2016.12.004

[B48] The Hindu (2020). *Coronavirus Lockdown — Over 80% of Students Depend on Mobiles for Learning: NCERT.* New Delhi: The Hindu.

[B49] The Social Media Family (2018). *IV Estudio sobre los usuarios de Facebook, Twitter e Instagram en España.* Available online at: https://thesocialmediafamily.com/informe-redes-sociales/ (accessed May 15, 2021).

[B50] Vilhelmson B. Thulin E. Elldér E. (2017). Where does time spent on the Internet come from? Tracing the influence of information and communications technology use on daily activities. *Inf. Commun. Soc.* 20 250–263. 10.1080/1369118x.2016.1164741

[B51] Wiley (2019). *The Use of Mobile Devices in Online Classrooms.* Available online at: https://edservices.wiley.com/mobile-devices-online-classrooms-use-demographic-higher-education/ (accessed December 4, 2020).

